# Opportunities and barriers for maternal nutrition behavior change: an in-depth qualitative analysis of pregnant women and their families in Uttar Pradesh, India

**DOI:** 10.3389/fnut.2023.1185696

**Published:** 2023-07-04

**Authors:** Neha R. Jhaveri, Natalia E. Poveda, Shivani Kachwaha, Dawn L. Comeau, Phuong H. Nguyen, Melissa F. Young

**Affiliations:** ^1^Behavioral, Social and Health Education Sciences, Rollins School of Public Health, Emory University, Atlanta, GA, United States; ^2^Doctoral Program in Nutrition and Health Sciences, Laney Graduate School, Emory University, Atlanta, GA, United States; ^3^Hubert Department of Global Health, Emory University, Atlanta, GA, United States; ^4^Program in Human Nutrition, Department of International Health, Bloomberg School of Public Health, Johns Hopkins University, Baltimore, MD, United States; ^5^Poverty, Health, and Nutrition Division, International Food Policy Research Institute (IFPRI), Washington, DC, United States

**Keywords:** antenatal care checkup, breastfeeding, calcium supplement intake, maternal nutrition, diet diversity, iron and folic acid supplement intake, weight monitoring, COM-B

## Abstract

**Background:**

Maternal undernutrition during pregnancy remains a critical public health issue in India. While evidence-based interventions exist, poor program implementation and limited uptake of behavior change interventions make addressing undernutrition complex. To address this challenge, Alive & Thrive implemented interventions to strengthen interpersonal counseling, micronutrient supplement provision, and community mobilization through the government antenatal care (ANC) platform in Uttar Pradesh, India.

**Objective:**

This qualitative study aimed to: (1) examine pregnant women’s experiences of key nutrition-related behaviors (ANC attendance, consuming a diverse diet, supplement intake, weight gain monitoring, and breastfeeding intentions); (2) examine the influence of family members on these behaviors; and (3) identify key facilitators and barriers that affect behavioral adoption.

**Methods:**

We conducted a qualitative study with in-depth interviews with 24 pregnant women, 13 husbands, and 15 mothers-in-law (MIL). We analyzed data through a thematic approach using the Capability-Opportunity-Motivation-Behavior (COM-B) framework.

**Results:**

For ANC checkups and maternal weight gain monitoring, key facilitators were frontline worker home visits, convenient transportation, and family support, while the primary barrier was low motivation and lack understanding of the importance of ANC checkups. For dietary diversity, there was high reported capability (knowledge related to the key behavior) and most family members were aware of key recommendations; however, structural opportunity barriers (financial strain, lack of food availability and accessibility) prevented behavioral change. Opportunity ranked high for iron and folic acid supplement (IFA) intake, but was not consistently consumed due to side effects. Conversely, lack of supply was the largest barrier for calcium supplement intake. For breastfeeding, there was low overall capability and several participants described receiving inaccurate counseling messages.

**Conclusion:**

Key drivers of maternal nutrition behavior adoption were indicator specific and varied across the capability-opportunity-motivation behavior change spectrum. Findings from this study can help to strengthen future program effectiveness by identifying specific areas of program improvement.

## Introduction

Maternal nutrition is a key contributor to maternal and child morbidity and mortality in many low- and middle-income countries (LMICs) (1). Pregnant women with poor nutritional status are more likely to experience adverse outcomes such as difficult labor, post-partum hemorrhage, poor birth outcomes, and increased morbidity and mortality (1–3). Several evidence- based interventions exist to improve maternal nutrition; however, there remain stark disparities in receipt of maternal health services across the globe (3, 4). In India, malnutrition is the leading risk factor for disease burden, contributing to 68% of the child deaths in 2017; out of all the states, the malnutrition Disability Adjusted Life Years was highest in Uttar Pradesh (5). In Uttar Pradesh, only 42% of women had four or more antenatal care (ANC) visits compared to 58% for all of India, and only 22% of pregnant women consumed iron and folic acid (IFA) for the recommended period of 100 days compared to 44% national average (6). These disparities and gaps in the implementation of evidence-based programs represent a public health challenge in Uttar Pradesh. There is a need to better understand the complex determinants of maternal nutrition-related behaviors in order to inform program improvements.

A global systematic review identified numerous factors that inhibit improvement in maternal nutrition in LMICs, such as household food availability, financial limitations, limited knowledge related to weight gain and food intake, and inadequate dietary counseling (7). Consideration of the broader socio-contextual factors that contribute to behavioral adoption, as well as aspects that affect women’s dietary practices during pregnancy, must be reflected in interventions to address critical gaps. The cultural context is an important consideration to understanding food norms, key actors (i.e., pregnant women’s husbands and mothers-in-law), and their decision-making power related to food decisions (i.e., the food purchased and meals cooked in the house) (8, 9). While much of the literature has focused on maternal attitudes, beliefs, and practices, family members can also be a powerful influencer of behavior change (10). In Nepal, for example, the mother-in-law had both a direct and indirect influence on the daughter-in-law’s food consumption, such as food quantity, types of foods, and the frequency of consumption (11). There is need for further context-specific research to understand role of family members in maternal behavior adoption during pregnancy.

In order to address the high levels of maternal malnutrition and poor program implementation in Uttar Pradesh, Alive & Thrive collaborated with International Food Policy Research Institute (IFPRI), IPE Global, and the Government of India to strengthen an integrated package of nutrition-intensified ANC services including maternal nutrition recommendations such as IFA and calcium supplementation, dietary diversity counseling, breastfeeding counseling, weight gain monitoring, and community mobilization (12). This study aimed to: (1) examine pregnant women’s experiences on key maternal nutrition-related behaviors during pregnancy (ANC checkup attendance, weight gain and monitoring, consuming a diverse diet, supplement intake) and breastfeeding intentions; (2) examine the influence of family members on these behaviors; and (3) identify key facilitators and barriers that affect behavioral adoption.

Our guiding theoretical framework for this study was the capabilities, opportunities, motivations, and behavior (COM-B) framework (13). The COM-B model of behavior is a valuable framework for identifying critical factors that need to be addressed in order for behavior change interventions to be effective. The COM-B model identifies three behavioral domains required to practice a specific behavior: (1) capability- the knowledge or skills needed to perform the behavior, (2) opportunity – the environment and/or social factors that make the behavior doable, and (3) motivation- aspects that can influence the intention and decision-making which drive a behavior. This framework has been widely used globally to examine complex human health behaviors including adherence to COVID-19 mitigation strategies, hand hygiene, and physical activity recommendations (14–19). In addition, the COM-B framework has been utilized to examine infant feeding practices and the associated beliefs to help inform and refine child nutrition interventions (20–22). Thus, the COM-B framework represents an ideal theoretical framework for providing novel insight on the complex components of maternal behavior change required for the successful implementation of the maternal nutrition program in Uttar Pradesh.

## Methods

### Study design, participants and setting

This qualitative study was embedded in a larger randomized-controlled impact evaluation of the Alive & Thrive interventions (12, 23). The larger study was conducted in the districts of Unnao and Kanpur- Dehat in Uttar Pradesh, and aimed to assess the delivery and impact of integrating a package of evidence-based recommendations (strengthening the provision of iron and calcium supplementation, counseling on dietary diversity, counseling on breastfeeding, and weight gain monitoring) within the existing government ANC services in 13 intervention blocks. One high-performing and one low-performing intervention block were selected after reviewing and analyzing the program data, which informed the sampling strategy for the interviews at the village level. In-depth interviews were conducted in 4 out of the 13 intervention blocks. Data was purposively collected in one high- and one low performing block in each of the intervention program districts in order to capture a range of program experiences. Twelve indicators were scored to determine whether a block was considered as “high-performing” or “low-performing” (14). For each block, one health subcenter (HSC), and 1–2 villages were selected (Supplementary Figure 1). For each village, 3 pregnant women within the 6 to 8- month of pregnancy from 1 to 2 households were randomly selected from the parent study registry list (24 pregnant women in total). Then, the pregnant women’s family members (13 husbands; 15 mothers in law- MILs) were identified for interviews to understand the family influence. The qualitative study was done to complement the parent study to obtain an in-depth understanding of program implementation and experiences.

### Data collection

Interviews took place from July 2019 through August 2019. In-depth interview guides were developed for pregnant women, MILs, and husbands in English, and later translated to Hindi (Supplementary Tables 1, 2). To ensure methodological rigor, during the development of the instruments, we received multiple rounds of feedback from our partners. The interview guides were pilot tested in Hindi in nearby blocks not associated with the study, which allowed for another round of modification before beginning data collection.

The interviews involved local research assistants who had a deep understanding of the local context. They assisted with interviews and reviewed the tools to verify that the original meaning of the questions was captured in Hindi. For quality control, the researchers randomly selected 10% of the transcripts and cross- checked them with the audio files to ensure accurate translation and quality transcription of the interviews. Between interviews, we debriefed with the research assistants as well as the other team researchers collecting interview data, and took time to reflect on field notes. At the time of analysis, we added memos to the interview transcription based on our observations, discussions during debriefs, and field notes. The guides covered several domains to help understand maternal nutrition behaviors and household dynamics and support, including daily activities, food habits, knowledge and attitudes toward the key indicators, experience with frontline workers (FLWs), and household dynamics.

Interviews were conducted in the participant’s residence. The interviews were audio-recorded and conducted in Hindi, varying in length from 25 to 60 minutes. Audio files were translated and transcribed verbatim into English.

### Data analysis and conceptual framework

**Figure 1:**
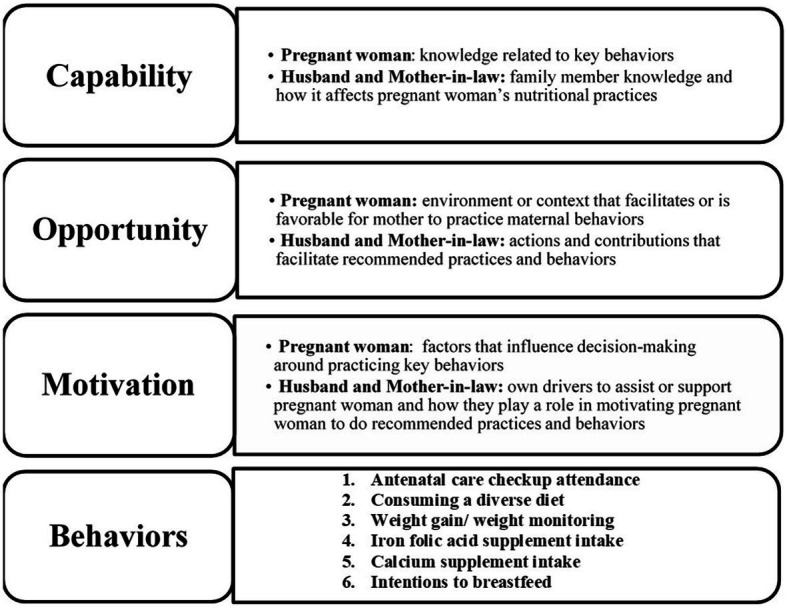
COM-B defined within the maternal nutrition intervention context in Uttar Pradesh.

Data was analyzed on MAXQDA2020 using a thematic analysis approach. The lead author was involved in directly conducting interviews as well as data analysis as part of her MPH thesis in Behavioral Sciences and Health Education. The lead author is fluent in English, understands Hindi, and has knowledge of many of the Indian cultural influences from her previous work experience, travel to India, and her own upbringing. Regular debriefing meetings were held with the research team and mentors to facilitate reflexivity, reduce potential bias and ensure analysis and conclusions were grounded in the data transcripts. The lead and co-author independently coded a select number of the family member interviews to develop a draft codebook, and then came to together to finalize the codebook before coding the remaining interviews, ensuring intercoder reliability and minimizing assumptions that could bias the data analysis. After the transcripts were coded, themes were categorized within the appropriate dimension of the COM-B conceptual framework (13). Figure 1 shows the three core components of COM-B (capability, opportunity, and motivation) as defined in the context of this study. Components that contribute to each indicator’s COM were then identified as either a facilitator or barrier. The lead and co-author then reviewed the balance of facilitators and barriers for each key behavior, and developed a summary of the data. Different interpretation of data between the researchers were discussed until consensus was reached about the meaning of the themes. The researchers also developed an overall subjective summary assessment of the level of intensity of facilitators and barriers for the capability, opportunity, and motivation components, which was informed by their assessment of how critical factors were to the behavior change of focus, as well as the prevalence of the facilitators and barriers for the behavior across the interviews (Supplementary Table 3). Based on the consensus of research and program team members, a qualitative summary was developed to denote low-, mid-, or high- level of facilitators or barriers for each component or to identify areas where there was insufficient data.

### Ethical approval

The Emory Institutional Review Board (IRB00111064) in USA and Suraksha Independent Ethics Committee (SIEC) in India approved study protocols. Oral informed consent was obtained before every interview. The study purpose, voluntary participation, and the effort to maintain confidentiality was stated in Hindi. Additionally, participants were told that they could skip questions or leave the interview at any point. Permission was also obtained before recording the interviews, and participants were informed that the recordings would be deleted after the completion of data analysis.

## Results

We examined key factors that influence women’s capability, opportunity, and motivation for each of the six maternal nutrition related behaviors (ANC checkup attendance, consuming a diverse diet, weight gain/weight monitoring, IFA supplement intake, calcium supplement intake, and breastfeeding intentions).

The COM components for each indicator are described in detail below, with overarching themes highlighted in italics. For each maternal nutrition behavior, key themes and supportive quotes for the identified facilitators and barriers within each indicator are summarized in Tables 1–6. There were no notable differences in factors influencing behavior change across high vs. low- performing blocks, so results were not divided by block type. In addition, Supplementary Table 3 provides an overall subjective summary assessment of the highly variable level of intensity of facilitators and barriers for the capability, opportunity, and motivation components to help to prioritize future program improvements.

**Table 1:** Facilitators and barriers for antenatal care (ANC) checkup attendance^1^.

Meta-theme	Barrier	Facilitator
Capability		
Level of knowledge	Receiving misinformation about timing of ANC checkup attendance: “*ASHA was saying in the 8 and a half month of pregnancy to get my [first] ultrasound done*.” (PW 23)	Family member awareness of services/tests provided during ANC checkups & knowledge of the importance of ANC examinations: “*Pregnant women should go to the community health center. [FLWs] will give proper care and properly perform her delivery, and PW will not have any problems since they will prescribe the medicine …ANC checkups are very important as it tells the exact position of the baby in the womb as well as the condition of the pregnant women*.” (MIL 1)
	Low level of family member knowledge/No knowledge related to ANC examinations or the importance: “*ASHA takes her. I do not know anything [about ANC checkups]. I do not ask [PW]*.” (MIL 4)	
Services provided by FLWs	Family members not aware of messages provided to PW: *“FLWs do not have any conversations in front of me so what can I tell you?”* (MIL 2)	
Opportunity		
Work burden	Daily schedule/duties that prevent PW from going to ANC checkup: *“I did not go there before 6 month of pregnancy because I have so much of work, but ASHA called me to go so I went with her.”* (PW 23)	
Family support	Family members do not accompany PW to the ANC checkups: *“I regularly ask my father-in-law and husband, but neither of them take me for check-up.”* (PW 20)	Family supports pregnant women taking/accompanying her to the health care facilities: *“Yes. I go with her [for her checkup]… Now it’s too far [for her] so I have to go.”* (Husband 4)
	Husbands live in other villages or out of the house due to work migration: “*I do not go with her. We have ASHA here, she takes her for checkup and my brother goes along with them, as I live out of the village*.” (Husband 5)	
Accessibility to ANC checkups	Inability to access ANC site due to location, lack of transportation, and weather conditions: *“I face difficulty in going from here to the hospital…I face difficulties in commuting and it’s very sunny, so I am not able to go.”* (PW 1)	Ambulance/Van transportation provided by FLW: “*I did not face any problem [going to the block hospital] because the ambulance comes to pick us up and drop us off*.” (PW 4)
FLW support	FLW dependence decreases family awareness/support: “*I do not go along with her all the time so I do not know. Anganwadi come here to call her so I send her to the Anganwadi center. Whatever is told, it is to her only. I do not know much*.” (MIL 3)	FLW support ensures that PW attend ANC checkup: *When they [FLWs] come to call her, she goes by herself as I am not at home, but if I am there I go with her*. (Husband 6)
Motivation		
Rationale	PW rely on FLWs to let them know when to attend ANC checkup: *“When [FLWs] call then I go [for the checkup] whenever they ask me to come, but when they do not call then I do not go…When they do not call I do my work.”* (PW 9)	
	Only go for checkup when they are experiencing a health issue: *Checkups? She goes for checkups when she has fever or cold… if she has fever she goes for checkups like that why will she go?”* (MIL 14).	Family members knowing the importance of ANC checkups: *“…those checkups are important if she will not go for checkup how we will come to know that how she is? That is why the checkups are very important.”* (Husband 9)
Experiences with the ANC checkups	Wait time/crowding at ANC checkup: *“I leave my home around 8 to 10 am in the morning and I have to be there till 3 pm. It always crowded there.”* (PW 17)	
Family support	No actions to support to pregnant women in relation to ANC checkups: *“She (ASHA) takes her. I do not know. I do not ask her.”* (MIL 4)	Intentions and action to help pregnant women: *“I go with her, and whatever they ask me to do, I do the same.”* (MIL 12)

1 PW, Pregnant woman; MIL, Mother-in-law; FLW, Frontline worker; ANC, Antenatal care.

**Table 2:** Facilitators and barriers for consuming a diverse diet^1^.

Meta-theme	Barrier	Facilitator
Capability		
Level of awareness/knowledge of dietary diversity and intake	Low/partial knowledge related to multiple food groups/food items important for a nutrient-rich diet: *“[FLW] said to eat nutritious diet to gain weight.”* (PW 1)	FLW counseling related to dietary diversity: *“[FLW tells me to eat] green leafy vegetables of five types, curd, milk, cottage cheese, fruits of yellow and orange color with pulp like banana, mango, carrots, some things according to season like pumpkin and jackfruit, and in breakfast boiled gram, red lentils, gram lentils and beans. Those who eat non-vegetarian food eat meat, but I am vegetarian, so she told me to drink 2 glasses of milk- because of this I had to add one glass of milk in my diet.”* (PW 24)
		Provided bowl/plate that indicate the quantities of the important foods: *“They [FLWs]…also gave me plate and bowl and asked me to have food in those utensils and prescribed the quantity of food to eat.”* (PW 2)
	Lack of nutrition knowledge from family members specific for PW: *“There is no difference in diet… we all will have that …whatever is cooked she will have that only.”* (MIL 7)	Family member having knowledge of benefits of consuming diverse diet, eating at multiple time points, and food hygiene: “*About eating- she should eat two times morning and evening. If she will eat four or five types of food according to her capacity then it will be good for her and her baby, too… She can eat rice and pulses, any of the green vegetables or meat- if she eats it will be good for her and she will be benefited.”* (Husband 9)
Opportunity		
Household food availability	Limited to food items available in the home: *“[FLW] tells me about good dietary habits, so whatever is available in the house, I eat that…”*(PW 3)	Family member is able to provide food items for the pregnant women: *“[FLW] call and give advice that what to eat and what not to eat…They tell that I should not eat such things which will damage my health and child’s health too, so she tells about foods like green vegetables, which my husband brings regularly, and milk we get in the morning which I use for my household and that I have myself as well. While coming back from work daily in the evening, my husband brings fruits like mango or papaya and grapes, pineapple, whatever he likes, he used to bring that. My husband does not let there be shortage in food items.”* (PW 2)
	Unable to purchase recommended food items due to financial strain: *“…She needs to eat fruits and drink milk. She should eat everything. If we do not have money we cannot give her to eat. But it’s important for her to eat proper food.”* (MIL 15)	
Food market availability	Market has limited food items: “*Yes, [FLW] said that I should be having fruits, milk, eggs and fish. Whatever I can eat among these…So whatever is possible for me, I have that. It is not possible to get fruits every day in the village. If it was a city, then it would have been possible*.” (PW 6)	
Food market access	“*The market is not open every day here. Only Saturday and Wednesday the market is open.”* (PW 19)	
Work burden	PW not eating foods due to busy schedule: *“I buy many things for her to eat but she does not eat them… my wife is not able to eat food on time due to work.”* (Husband 11)	Family member able to assist with tasks: *“I help her in cooking good food. Whatever work she is not able to do I will do.”* (MIL 15)
Motivation		
Awareness of benefits of consuming nutritious diet		Understanding positive impact of diet on mother/child: *“I feel like if I will take care of my diet, the baby will keep healthy and I will also not get any problem, so I will not require medicine in future if I will take care of diet.”* (PW 2)
Family support	Family members potentially deliver messages in forceful way: *“[PW name] does not feel like eating, but still I forcefully make her eat for her baby.”* (MIL 12)	Family members providing reminders and support of key behaviors because of benefits to PW: *“One person should be there with her always it should also be seen that she has eaten, how many times in a day has she eaten, she has water to drink* etc*… when the family members take care of her then only she will be able to do anything.”* (Husband 12)
		Perceived responsibility to assist PW with consuming nutritious diet: *“It is my responsibility as I bring everything for her, to make her eat all that is the responsibility of my mother as I go out to work.”* (Husband 10)
Food preferences	Focus on eating foods that are liked but not necessarily the recommended food items: *“Nutritious food is important, but she eats whatever she likes.”* (MIL 9)	
Pregnancy/medication related effects	Nausea/vomiting limited food consumption: “*Yes I had some difficulties [eating]- I did not like the food since I was vomiting and had nausea*.” (PW 1)	

1 PW, Pregnant woman; MIL, Mother-in-law; FLW, Frontline worker; ANC, Antenatal care.

**Table 3:** Facilitators and barriers for weight gain and monitoring^1^.

Meta-theme	Barrier	Facilitator
Capability		
Level of knowledge	Not informed on the importance of weight monitoring*: “I do not know how much my weight is. ASHA only knows about my weight, I do not know, I only stand up on the weighing scale.”* (PW 8)	Knowledge of link between dietary intake and weight gain: *“Yes, when they measure my weight, at the time [FLW] gave information regarding proper diet and said if I eat properly that I will gain weight.”* (PW 19)
	Low knowledge related to PW weight gain monitoring or its importance: “*A ma’am used to come and monitor the weight so [PW] must know why weight should increase-I do not know.”* (Husband 2)	Family member knowledge of the importance of weight gain/weight monitoring: “*Yes, they [FLWs] told us that weight gain monitoring should be done every month because through this it will come to know whether there is any deficiency in baby or not*.” (MIL 5)
Opportunity		
ANC visits		Weight monitoring as part of home visits: *“I went to the [ANC site] for immunizations and met [FLW] there, and once she came to my home for weighing my weight- she used to come often.”* (PW 17)
ANC checkup site	Issues at the ANC checkup site with weighing scale: “*I visited twice but they did not measure my weight. Yesterday, they were telling that in the [ANC site] that there is no weighing machine and that is why they are not measuring weight.”* (PW 23)	
Motivation		
Importance for maternal/child health		Recognition of importance of weight gain to avoid negative effects: *“Yes, [FLW] says that, weight should not decrease, otherwise you and your baby both will have problem, and if you are having good diet, this indicates that your baby is in good health and you are also having good health.” (PW 2)*
Family support	No information to inform providing support PW for weight gain and monitoring: *“She used to go alone- I do not go along with her all the time so I do not know…Whatever is told, it is to her only. I do not know much.”* (MIL 3)	

1 PW, Pregnant woman; MIL, Mother-in-law; FLW, Frontline worker; ANC, Antenatal care.

**Table 4:** Meta-themes and facilitators and barriers for iron folic acid supplement intake^1^.

Meta-theme	Barrier	Facilitator
Capability		
Level of knowledge		Knowledge of how to take tablets: *“[FLW] also told how to take the iron tablets, as I did not feel good once I ate iron tablet, so she told me to have it with lemon water in the evening. Now I feel better when I take the tablet that way.”* (PW 9)
Family member awareness of PW’s intake of IFA	Low awareness of side effects that PW may experience: *“There is no side effect of the medicines, in fact, they are only benefitting from it.”* (MIL 1)	
Opportunity		
Accessibility/availability of IFA supplement		Access to tablets through FLW: *Yes, I go and tell [ASHA] that medicines are finished, then she comes and gives them to me.”* (PW 24) Family getting IFA: *“We bring those [IFA] tablets for her.”* (MIL 9)
Motivation		
Rationale	Not aware of importance/benefits of IFA: “*My daughter in law can only tell you the benefits of this tablet … As she ate the tablets, she can only tell that what happens in her body. If I have taken it then I would have told you the effects of those tablets*.” (MIL 7)	Knowledge of benefits of IFA supplements for mother (during delivery) and baby: *“…She told me that iron tablets help to increase the blood level and that I will not feel dizzy and weak, and also the baby will be healthy and fine… She told me about the advantages of the pill so I take it.”* (PW 9)
Side effects	Side effects that deter PW from taking tablets: *“No, I had 4 to 6 tablets but then, I started feeling nausea so I stopped having it.”* (PW 23)	“*Earlier when I was having iron pills, I used to vomit. Then [FLW] told me to take medicine with lemon water, now take it with lemon water after my evening meal before going to bed. Now I do not have any problem in having iron pill.” (PW 8)*
Family support	Sharing partially incorrect messages for IFA intake: *“I ask her to drink it with milk. If she is not able to drink with milk then she should drink it with lemon water.”* (MIL 15)	Family members support PW: *“I remind her to have tablets.”* (Husband 8)
	No perceived role with supporting IFA intake: *“How can I help her with [taking IFA]?”* (Husband 5)	
	Inconsistent support: *“I used to tell her to eat and remind her to take medicine. Now, whether she has or not I do not keep a check.”* (MIL 4)	

1 PW, Pregnant woman; MIL, Mother-in-law; FLW, Frontline worker; ANC, Antenatal care.

**Table 5:** Facilitators and barriers for calcium supplement intake^1^.

Meta-theme	Barrier	Facilitator
Capability		
Level of knowledge	Family members unclear on distinction between medications: *“She takes medicines… one is red in color and other is white in color.”* (MIL 14)	Knowledge of how and when to take tablets: *“Yes, I get the iron and calcium tablets from there, and I was also told how to consume them, that the red tablets should be eaten at night after the meals at sleeping time, and the white one in the morning after the meal. So, one tablet in night and one in daytime.”* (PW 2)
	Unclear on benefits specific to calcium: “*She said that tablet will give energy and strength to her and also helpful in relieving pain if she has any pain.”* (MIL 5)	Family member having knowledge of benefits: *“Yes, they [FLWs] gave information that calcium will strengthen bones and IFA tablets help iron levels increase.”* (Husband 8)
Opportunity		
Access to calcium supplements	No availability of calcium tablets: *“I could not get calcium tablets…It is not available anywhere. There is no distribution anywhere.”* (PW 6)	
	Inconsistent availability of calcium tablets: *“I got calcium tablets initially, but after that they told me calcium tablets are not available, so I only take IFA tablets.”* (PW 19)	
	Issues purchasing from pharmacy/medical store: *“For one month I have not had [calcium pills] because there is a money problem. My husband had to buy it from a private pharmacy. I could not get it from [ANC checkup site]…I did not get white calcium medicine, I only got red [iron] medicine.”* (PW 8)	Family member support in getting calcium tablets when not available through ANC: *“Yes, I bring those for her- she gets the iron tablets [from ANC] but when calcium is less, I get it from medical store.”* (Husband 9)
Motivation		
Rationale		Knowledge that calcium is beneficial for baby’s health*: “[FLW] told me to have calcium tablets then my bones will become strong and by IFA tablets your blood as well as baby’s blood will increase, so if I will be healthy then my baby will attain good health.”* (PW 14)
Family support		Intentions and action to help pregnant women: *“After eating she has to take the [calcium] tablets…if [PW] does not do it then I call her to ask her whether she has done or not so she tells me, but if I feel I cannot rely on her, then I usually ask my mother whether [my wife] has eaten or not then my mother says that yes she has eaten. I rely on her.”* (Husband 12)

1 PW, Pregnant woman; MIL, Mother-in-law; FLW, Frontline worker; ANC, Antenatal care.

**Table 6:** Facilitators and barriers for breastfeeding intentions^1^.

Meta-theme	Barrier	Facilitator
Capability		
Level of knowledge	Inaccurate exclusive breastfeeding knowledge: “*When baby becomes 6 days old we start giving [goat, buffalo and cow’s milk] with breastfeeding.”* (PW 23)	Exclusive breastfeeding knowledge*: “… no other milk should be given only mother’s milk.”* (PW 24)
		Knowledge of breastfeeding practices: *“I was told how often the child should be breastfed. Ten minutes or half an hour. Whenever child is hungry or opens mouth, breastfeed the baby.”* (PW 1)
	Cultural practices and feeding baby after delivery: *“After one hour of birth, milk should be provided and jaggery. I provide boiled buffalo milk with jaggery…I smear jaggery in the foot of baby then give bath to the baby and the cut the umbilical cord and give goat’s milk. Within one month after birth, mother breastfeeds the baby. And when baby attains 6 months we give him powder milk.”* (MIL 9)	Family member knowledge of early initiation of breastfeeding: *“During the first hour after birth, one should only breastfeed the baby. Nothing other than that.”* (MIL 5)
		Family member knowledge related to exclusive breastfeeding: *“For the first six months the baby will have breast milk-after that we can give him different types of nutritious food.”* (Husband 6)
Knowledge of addressing issues related to breastfeeding	*If breast milk does not secrete, then a woman should give goat’s milk.* (PW 22)	Actions to take if PW has problem with breastfeeding: *“If she will face any problem [with breastfeeding] then we will take her to the doctor.”* (MIL 11)
	Family members having inaccurate knowledge of actions to take if PW is having issues breastfeeding: *“If the mother is not producing milk then the cow’s milk or horse’s milk can be given to the baby when he will drink then with the help of needle, very hygienically, baby can be fed the milk. The baby will feel relaxed, then she will automatically feed the mother’s milk. The child has to be kept very clean, the clothes and everything else should be properly clean and hygienic.”* (Husband 12)	
Opportunity		
Family support		Intentions to support the PW breastfeeding: *“Suppose mother is busy in some other work, and then I look after baby I feed baby with powder milk (top milk) and if she breastfeeds the baby then I do other things like massage of the baby or change baby’s diaper.”* (MIL 1)
Motivation		
Rationale		Knowledge of benefits of breastfeeding*: “Because of breastfeeding, if the child suffers from something, he/she will not get affected by it.* (PW 2)
		Family member knowledge of benefits of breastfeeding: *“If the child does not drink mother’s milk then how will he connect to his mother (talking about emotional connection)? It is a famous saying that the child will leave his parent when he will grow up, that is why the elders say that to breastfeed the child then only the child will have some feelings towards and connections with his parents. You can skip giving cow or buffalo’s milk but mother’s milk is very necessary and nowadays women do not breastfeed the baby because it hurts them.”* (MIL 3)
Family member responsibility related to breastfeeding	*“How can I contribute [to my wife’s breastfeeding]- it is the mother’s responsibility to feed the baby and keep the he/she comfortable.”* (Husband 6)	Notifying mother to feed baby: *“If my baby is crying because of hunger and my wife is busy in her work then I will tell her to breastfeed the baby.”* (Husband 7)
	Providing support only if PW seeks help: *“If she will stay here then we will all tell her and help her. See, if she tells me that she is having any problem then only I can help her but if you are not informing me about anything then how can I help?”* (MIL 3)	

1 PW, Pregnant woman; MIL, Mother-in-law; FLW, Frontline worker; ANC, Antenatal care.

### Antenatal care (ANC) attendance

#### Capability

Despite women having some level of *awareness and knowledge of ANC checkups*, most pregnant women and family members did not report knowing the importance of early and frequent health visits during pregnancy, and instead were more likely to go for an ANC visit only if they were experiencing a health issue (Table 1). Women were aware of the ANC services offered and/or received during their past visits, including blood and urine tests, sonograms, and supplements as well as dietary and hygiene information. The family members who attended ANC checkups with their wife/ daughter-in-law were also able to recall FLW advice on key behaviors and the importance of services offered during ANC visits. Conversely, capability was lower among family members who did not accompany pregnant women to the ANC check up.

#### Opportunity

High household *work burden* was a barrier to attending ANC checkups. In some cases, women had family members who would help with household tasks, which mitigated this barrier to ANC checkup attendance. However, women who did not have *family member support* would often not go for their ANC checkups on their own. Family members who provided low levels of support placed the responsibility to support solely on the FLW. A few MILs expressed they did not accompany pregnant women to their ANC checkups either because the FLWs asked them to stay at home or the pregnant women did not directly request their support. Many husbands reported not being at home or in the village during the times when women go for the checkup. Another factor that affected opportunity for ANC checkup attendance was women’s perceptions on *accessibility of ANC checkup site*. Women cited that hot weather and distance from the community or primary health centers made going for checkups challenging. However, accessibility barriers were overcome for some women who shared that the FLWs provided a van or ambulance to take them to and from the health center. *FLW support* through home visits also emerged as an important facilitator as it allowed for pregnant women to continue with their daily work routine and responsibilities and prevented them from discomfort typically experienced en route to the ANC site.

#### Motivation

Overall, there was low reported motivation for attending ANC checkups among pregnant women. Women shared they were likely to go for a checkup if they were facing a health issue that they felt required immediate attention, or if a FLW instructed them to go that day and offered to either accompany them or provide the transportation for them to do so. Another key facilitator included home visits from FLWs, as they reminded women to attend their checkups. In contrast, infrequent FLW visits resulted in missed checkups during pregnancy.

A key contributing factor to the *rationale-* or reason that facilitated the ANC checkup attendance- included pregnant women and/or their family members having awareness of the need and importance of ANC visits. For women who had been to the ANC site before, their *past experience with ANC checkups* also influenced the motivation to attend visits. Women expressed that long waiting times and crowding during checkups, lack of respect from health care professionals, and commute discomfort all decreased their motivation for attending future checkups.

### Consuming a diverse diet

#### Capability

Among the participants there was a high overall *awareness and knowledge of dietary diversity* recommendations during pregnancy (Table 2). Both pregnant women and family members acknowledged the importance of a nutritious diet for the health of the mother and child. The majority were able to note specific food items that should be consumed during pregnancy, but only a few mentioned multiple food groups such as dark green leafy vegetables, grains, pulses, etc. However, a challenge women faced in meeting these recommendations was lack of knowledge surrounding substitute or alternative foods of similar nutritional value that could be consumed when they cannot eat the recommended items due to seasonal availability, dietary restrictions (i.e., do not consume any meat or eggs), or cost. Additionally, some of the women reported not knowing the quantity of food they should consume, unless they received the bowl or plate for measuring while they were learning about dietary intake.

#### Opportunity

The opportunity for pregnant women consume a diverse diet during pregnancy was influenced by *household food availability*, and local *food market affordability*, *availability, and access*. Women and family members frequently reported that financial constraints restricted their ability to purchase certain foods. A key complaint that some women had was that their markets had limited food items or that the market was difficult to access (not open every day; travel limitations for women). Women reported that family members, in particular husbands, were primarily responsible for purchasing foods from the market, and if husbands were aware of the nutritious food items, they would buy foods consistent with the FLW dietary recommendations. Women also discussed that high *work burden*- particularly taking care of household responsibilities and caring for children- prevented them from eating properly or missing meals.

#### Motivation

Motivation to consume a nutritious diet was primarily driven by perceptions of the *benefits of consuming a nutritious diet* and belief that eating a proper diet will have a positive impact on the mother and child. *Family support* was also a strong motivator where family members provided reminders and encouragement for the pregnant woman to eat the recommended foods when they felt the behavior would have a positive impact on the child. There were mixed perceptions on roles and responsibilities within household on maternal diet; in some households the burden was placed solely on the pregnant woman, while in other households the husband and MIL felt the responsibility for ensuring a healthy diet. However, in some cases, the family member’s concern for the health of the mother and baby was conveyed negatively through scolding and reprimanding. In addition, individual barriers such as *food preferences* (likes and dislikes) and *nausea* were barriers to women meeting dietary recommendations.

### Weight gain and monitoring

#### Capability

There were mixed *levels of knowledge* reported on weight gain during pregnancy (Table 3). Few women knew their current weight and how much weight they should gain during pregnancy. Women typically shared that FLWs informed them on how often they should get their weight checked. Some women were told they should be gaining weight, but did not know why this is important or how to meet weight gain recommendations through diet. One misperception that pregnant women expressed was that there were potential negative implications of her weight gain on the child’s health because additional weight would put pressure on the baby.

Most family members reported not knowing why weight should be monitored during pregnancy. However, the family members who accompanied women to ANC checkups were aware of recommendations and were able to share the importance of increased food consumption and diversity to gain weight during pregnancy.

#### Opportunity

Some women were weighed at the ANC checkup sites while others were weighed during home visits which mitigated any issues related to getting to the ANC site. However, weight monitoring was intermittent as women did not go frequently for their checkups, or there was inconsistency with the function of the site’s weighing scale during their ANC visits. Weight monitoring was often a missed opportunity to talk about nutrition-related topics as most women who were weighed reported simply having their weight taken without any further discussion.

#### Motivation

Data related to the motivation toward weight gain/weight monitoring was not salient. Although there were a few pregnant women, husbands, and MILs who recognized the *importance of weight gain for maternal/child health*, it was not clear whether it was a driving factor that influenced their attention toward weight gain through the pregnancy period. *Family support* was limited as family members did not discuss any role or responsibility to help pregnant women to gain and monitor their weight.

### IFA supplement intake

#### Capability

Pregnant women’s capability level related to IFA supplements (Table 4) was high overall with their *knowledge surrounding intake*, specifically the time of day for taking the tablets, how to consume them to minimize side effects, and the importance of IFA for her own health as well as for her child. However, only a few women were able to share the specific benefits of taking IFA supplements during pregnancy. Some family members – particularly MILs- had an understanding that consuming IFA was important and were generally aware of whether or not the pregnant woman was taking IFA, so they were more likely to provide support for her IFA consumption. Husbands’ awareness involved knowing where the tablets were distributed, and that the doctor or FLW was the person who held the knowledge related to consumption. However, husbands did not see themselves having any role with supporting women to take IFA. Only a few family members had knowledge related to the tablets themselves and the associated side effects, so majority believed women were not experiencing any issues with consumption. Additionally, there was conflicting knowledge related to the best practices advised by family members versus FLWs. For instance, some family members said to take tablets with milk instead of with lemon water, as recommended.

#### Opportunity

Since all pregnant women were provided the tablets either through home visits or during their visit to the ANC checkup site, there were no noted barriers to IFA access or availability for opportunity. Supply and *access to the tablets* were not an issue with IFA among participants.

#### Motivation

*Knowledge of anemia* and its effects, as well as the benefits of consuming IFA for the body and her baby facilitated and motivated pregnant women to consume IFA. However, *physical discomfort* associated with taking the tablets negatively affected motivation for some women to take IFA. Women who took the supplements reported that feeling nauseated or flushed around the time of taking the tablets interrupted the consistency of their consumption. Reminding the pregnant woman to take the tablets, as well as bringing her a beverage to help consume the tablets, were examples of how *family members provided support.* However, not all family members were noted playing a role in her IFA intake regularly or accurately.

### Calcium supplement intake

#### Capability

While there was some accurate knowledge shared in interviews when discussing calcium intake, the overall level of knowledge was not consistent amongst participants (Table 5). While most pregnant women were able to recall aspects like *timing of day for consumption*, how to take the tablets, or the importance of taking these tablets, this was not the case for family members. Family members had limited awareness of calcium supplements and pregnant women’s intake of these tablets. Often, husbands and MILs had difficulties in *differentiating between iron and calcium tablets*. When some family members were asked about calcium, they would refer to consumption of other medicines or the supplement, which made it unclear whether their information applied to calcium supplement or other tablets.

#### Opportunity

Opportunity emerged as a key determinant of calcium intake due to *inconsistent availability and provision* of calcium tablets in health care facilities, which resulted in the need for women or family members to purchase them from pharmacies or medical stores. Since calcium tablets were not provided at the time of the ANC visit, *not having financial means to buy the tablets* emerged as a barrier for calcium supplement access and therefore calcium intake.

#### Motivation

Women who had a general understanding of the importance of *calcium intake to benefit her and her baby* reported consuming the tablets when they had access to them. *Family support* was low as their was limited understanding of the importance of consuming calcium supplements. Although two husbands with high involvement shared that they tried to remain involved in monitoring their wives’ frequency of intake, this was not noted in any of the other interviews. The support that MILs mentioned providing was general encouragement to take supplements, but this was not specific to calcium.

### Breastfeeding intentions

#### Capability

The *timing of messages related to breastfeeding practices* was a key determinant of breastfeeding capability (Table 6). Although women were in the 7th or 8th month of pregnancy, one-third of women had low level of knowledge as they *did not receive any breastfeeding counseling*. Women’s knowledge primarily consisted of time intervals for feeding and frequency of breastfeeding. While most women knew exclusive breastfeeding should continue for 6 months after birth, a few women mentioned that babies could have water, other types of milk, and foods during that period. Similarly, family members mentioned offering the baby cow’s (or another animal’s) milk, jaggery (sweetener derived from sugar cane), honey, and other liquids or foods early on after the baby’s birth, which are culturally informed practices but interfere with exclusive breastfeeding. Additionally, some women and family members felt that other forms of milk were acceptable as an alternative to breastmilk if the pregnant woman was *having issues breastfeeding*. Level of knowledge about early initiation of breastfeeding varied among husbands and MILs. Some family members had knowledge of the time frame for exclusive breastfeeding and mentioned the benefits breastfeeding has for both the baby and the mother. Family members also expressed confusion when asked about how the baby should be fed when he or she becomes sick. Women who had given birth in the past shared that they received information related to early initiation of breastfeeding at the community health center, revealing that *institutional delivery* was also a point of time to receive accurate information on breastfeeding. Related to knowledge of providing support while the mother is breastfeeding, a few family members mentioned they would help to improve the woman’s diet as they accurately associated quality of diet as a way to provide support related to breastfeeding.

#### Opportunity

Information related to breastfeeding opportunity was limited given that women and their family members were interviewed during the pregnancy period and not after delivery when breastfeeding would begin, so responses highlighted potential aspects of opportunity. While most husbands’ interviews did not reveal information on how they could *provide support* his wife while she is breastfeeding, MILs anticipated difficulties and were able to identify various reasons where the mother may have problems, such as the baby excessively crying, painful breasts, and other issues that may require treatment. Although most husbands and MILs said that the mother is primarily responsible for breastfeeding, a few MILs and one husband mentioned they would help the pregnant woman with tasks to give her time to breastfeed.

#### Motivation

The benefits of breastfeeding (i.e., improving the baby’s development) were mentioned by some women, husbands, and MILs. Husbands had limited knowledge of breastfeeding, and most *did not perceive any role to support* their wives, viewing this practice as solely a women’s role. However, few husbands and MILs mentioned waking the mother up if she is asleep and letting her know that the baby is crying as ways to support breastfeeding.

## Discussion

Our qualitative study explored the facilitators and barriers that contribute to the capability, opportunity, and motivation to adopt key maternal nutrition behaviors, including: diet diversity, ANC checkup attendance, weight gain and weight gain monitoring, IFA supplement intake, and calcium supplement intake, as well as the intention to breastfeed after delivery in Uttar Pradesh. The level of capability, opportunity, and motivation present varied across the six promoted maternal nutrition behaviors. Overall, the findings demonstrated the following: ANC checkup attendance was low in motivation due to the lack of understanding of the importance for going to checkups routinely during pregnancy. Participants described key drivers that make it more favorable for pregnant women to adopt the recommended nutrition behaviors, such as having an ambulance take women for their ANC visit to counter transportation and accessibility issues, FLWs conducting home visits that allow for women to continue with daily responsibilities and still receive health advice, and family members providing encouragement and logistical support necessary for her to follow FLW advice. While capability did not appear to be a limiting factor for dietary diversity/ intake, many structural issues (financial strain, high food costs, limited food availability in markets, and accessibility) affected opportunity and inhibited behavioral change. Both facilitator and barriers were present in capability and opportunity for weight gain/ weight monitoring, but motivation was lacking. While there were no reported issues with getting IFA tablets, lack of opportunity was the largest barrier for calcium supplement intake due to supplement shortages. Breastfeeding capability was a key limiting factor with many women reporting inaccurate messages and misperceptions. This research provides insight into the overall key maternal nutrition intervention results and suboptimal practices previously reported in this population (12, 23).

The COM-B model has been widely used to identify where attention and efforts are necessary within a behavior intervention to improve implementation and effectiveness (13, 15, 20, 21, 24). The Health Belief Model (25), the Transtheoretical Model (26), and the Theory of Planned Behavior Model (27) are also commonly cited health behavioral change models to used explain underlying determinants of behavioral adoption, decision-making, and outcomes. The Health Belief Model looks at an individual’s perceived susceptibility of a health risk or illness, and the perceived benefits and barriers of practicing a health behavior, but it overlooks the influence of an individual’s environment and operates on the assumption that individuals have the same information to move forward with a behavior. While the Transtheoretical Model is able to capture the stages of decision-making to ultimately create behavior change, it is designed with the assumption that the thought process is linear, and is also unable to reflect the role that contextual factors (such as socioeconomic status) have in the ability for individuals to follow through on the behavior of focus. The Theory of Planned Behavior takes beliefs, intentions, and an individual’s ability into consideration, as well as the influence of social norms, on completing a behavior, but it does not take into account an individual’s opportunity or if they have the necessary resources to do so. As the COM-B framework (13) encompasses the contextual factors, contributors to decision-making, and notes the interactive components that play a role in behaviors, it was a valuable tool to help identify factors that contribute to maternal nutrition practices through the three distinct dimensions of behavior change: capability, opportunity, and motivation. Defining the facilitators and barriers for each behavior within the three dimensions deepened our understanding of important individual, interpersonal, and contextual considerations essential to maternal nutrition practices during pregnancy and for guiding future improvements in program implementation.

*Capability* was a critical first step for enabling maternal nutrition behavior change. Among the key behaviors, capability was particularly notable for both pregnant women and family members in relation to consuming a diverse diet. Women and family members shared their knowledge of food items that are beneficial for the mother to consume during pregnancy, which is important for informing decision-making related to daily dietary intake (28). Customization of messages by FLWs to ensure guidance both met nutritional needs and considered what was locally available at the market or in season was important. Similarly, women had shared accurate knowledge when discussing IFA supplement intake and did not have difficulty reporting the importance of taking the supplement, the time of day to take it, and ways to reduce side effects; these are key aspects that underlie the consistent IFA intake that are necessary for its effectiveness (29). Capability around breastfeeding was low among participants in this study compared to the other behaviors. Interviews were conducted during pregnancy and many women had not yet received breastfeeding counseling. Receiving counseling messages at or close to birth rather than during the ANC period is a missed opportunity and many studies have demonstrated the importance of both time periods for effective behavior change (3, 30). Late ANC checkups result in less than four ANC checkups during the pregnancy period, and have implications on other key behaviors; women not only miss counseling related to key behaviors, but are also unable to take IFA for at least 180 days as recommended by the World Health Organization. Studies conducted in Uttar Pradesh as well as other parts of India suggest that maternal knowledge contributes to higher self-efficacy and increased likelihood of breastfeeding, but cultural practices that are believed to benefit the infant (i.e., feeding jaggery within hours of birth) are contradictory to correct practices and are encouraged by MILs (10, 31). As MILs tend to be the authoritative female in the house with say in nutritional practices and decision-making, it is essential for them to be actively engaged when these practices are discussed (11).

However, capability did not guarantee that all pregnant women adopted these behaviors, as seen when examining the *opportunity* dimension. When looking at opportunity of consuming a diverse diet, the level of knowledge shared for consuming a diverse diet was only a facilitator when women had the ability to obtain those foods. Participants mentioned that seasonality, food availability in the market, and food affordability were barriers they encountered when trying to follow the dietary advice provided. These key barriers are consistent with other states within India and various LMICs, and limit the likelihood of women meeting minimum diet diversity (32–35). Men primarily made purchases and money-related decisions and some husbands mentioned how they used their nutrition knowledge to purchase foods that benefitted the wife’s health during pregnancy when finances allowed. Most women in this study understood the importance of IFA and did not report issues in obtaining IFA, which contrasts with findings in prior literature in Africa and Southeast Asia that has reported limited knowledge on the importance of taking IFA supplements, supply chain issues, and lack of receipt of IFA during pregnancy (36, 37). Conversely, in this study, calcium supplement intake was significantly hindered due to limited access to calcium tablets and required more steps (i.e., going to the medical store to purchase tablets) that further decreased likelihood of consumption, resulting in disruption or inability of consistent intake. Early and frequent ANC visits provided a critical opportunity for counseling and receipt of services for several of the maternal nutrition indicators (receiving advice on nutritious foods and diverse diet important for the baby, having weight checked, obtaining supplements, etc.) all of which have the potential to impact birth outcomes (38). The opportunity to attend ANC checkups was dependent on family support and has been reported in prior literature (39).

*Motivation* was a limiting factor across maternal nutrition indicators. Overall, both individual and family member capability were influential on motivation for behavior change. Specifically, motivation was found to be an important component with supplement intake, and knowledge of the benefits to the mother and child of taking the supplements contributed to their intentions to take the tablets. The knowledge of benefits to the mother and child, as well as prevention of anemia, were key drivers for IFA supplement intake, so having FLWs emphasize those aspects during counseling is crucial (40). Family members who provided reminders for women to take tablets contributed to supplement consumption, as noted in other studies (41). Limited motivation related to weight gain may have been related to inconsistencies in understanding the importance of weight gain over the pregnancy period and to the quality (or lack) of counseling (42).

One strength of this study was the analysis of the data through the COM-B framework alongside the involvement of different family members, which allowed for holistic understanding the family dynamic influence and the important contextual factors that surround each of the maternal nutrition indicators. Additionally, the qualitative analysis process involved two researchers coding independently initially before coming together to develop the codebook and ensured intercoder consistency. The interview guides also covered a variety of topics, which provided specific insights on numerous aspects of the household level. However, this also restricted the ability to ask multiple questions about one or two indicators in order to avoid participant fatigue. In exploring the capability aspect, the nature of the questions regarding key messages from FLWs was partially dependent on the level of recall by each pregnant woman and family members. In turn, the information shared may not be an accurate representation of the information provided during the visits. When researchers asked questions about their interactions with the FLWs, and whether or not they were told or instructed to help them with the performance of key behaviors, many women could have been worried about implications of giving an honest response, i.e., getting the FLWs in trouble, which may have resulted in sharing limited details when responding to the questions about their interactions with them. Additionally, social desirability bias was a potential limitation. Since the interviews were conducted in the pregnant women’s household, potential for others to overhear what the participant was sharing, or have a family member walk in during the interview also may have been a concern when talking about herself and her family.

In order to get a clearer picture of all factors that influence and underlie the adoption of behaviors, it is essential to combine insights from the pregnant woman, husband, MIL, and/or other key actors who play a role in the process. For example, previous studies have shown that looking at FLWs and pregnant women perspective side by side can reveal inconsistencies that can clarify some of the barriers reported among the pregnant women (43). Thus, considering a holistic analysis can reveal the significance and the weight of the facilitators and barriers that emerged during the interviews with the pregnant women, and inform how the household unit acts on recommendations of key behaviors.

This study focused only on the husband and MIL, but some of the pregnant women responded that other family members, like the sister-in-law, provided important support that differed from the husband and MIL, since she was often of the same or similar age, and in some households, also pregnant. Involving more family members can enhance the behavioral adoption process for the pregnant women, and also shift the way in which support is provided within the household. Future research can also be done to understand how programs similar to this have a direct influence on family member’s behaviors and practices. Since husbands and MILs are socially viewed as authoritative figures, considering the influence from an equivalent peer could be explored further. Using a behavior change theory during programmatic development and analysis can allow for an in-depth understanding of the reasons behind decision-making, and the role of socio-contextual components during the behavior adoption period (44). This study only applied a portion of the framework developed by Michie et al. (13) but next steps could include understanding all the factors that are included in the behavior wheel such as policy level influence.

Our qualitative study, using the components of the COM-B framework, provided novel insight into the key facilitators and barriers that affect the practice of key maternal nutrition behaviors: diet diversity, ANC checkup attendance, weight gain monitoring, and IFA and calcium supplement intake within the context of a large ongoing maternal nutrition intervention in Uttar Pradesh. Our family centric approach provides a unique and more complete look at the household level. Information will contribute to implementing targeted and comprehensive programmatic actions to improve the implementation of maternal nutrition interventions in the region.

## Data availability statement

The raw data supporting the conclusions of this article will be made available by the authors, without undue reservation.

## Ethics statement

The Emory Institutional Review Board (IRB00111064) in USA and Suraksha Independent Ethics Committee (SIEC) in India approved study protocols. Written informed consent for participation was not required for this study in accordance with the national legislation and the institutional requirements.

## Author contributions

MY, NJ, NP, SK, and PN designed the research, under the mentorship of PN, MY, DC, and SK. NJ led the interviews with the pregnant women and their husbands and had primary responsibility for the final content. NP led the interviews with the mothers-in-law. NJ and NP analyzed the data. MY, NJ, NP, SK, DC, and PN aided in interpretation of data and reviewed and revised the manuscript. All authors read and approved the final manuscript.

## Funding

This work was supported by the Bill & Melinda Gates Foundation (through Alive & Thrive, managed by FHI Solutions) and Emory University.

## Conflict of interest

The authors declare that the research was conducted in the absence of any commercial or financial relationships that could be construed as a potential conflict of interest.

## Publisher’s note

All claims expressed in this article are solely those of the authors and do not necessarily represent those of their affiliated organizations, or those of the publisher, the editors and the reviewers. Any product that may be evaluated in this article, or claim that may be made by its manufacturer, is not guaranteed or endorsed by the publisher.

## Abbreviations

ANC: Antenatal care
COM-B: Capability-Opportunity-Motivation-Behavior framework
FLW: Frontline worker
IFA: Iron and folic Acid
MIL: Mother in-law
