# Supplementary material for: Opportunities and barriers for maternal nutrition behavior change: an in-depth qualitative analysis of pregnant women and their families in Uttar Pradesh, India
Source: Front Nutr. 2023 Jul 4;10:1185696. doi: 10.3389/fnut.2023.1185696 (PMC10352842; doi:10.3389/fnut.2023.1185696)
Supplement: Supplementary file 1 [file Data_Sheet_1.docx]

Supplementary Material

Opportunities and barriers for maternal nutrition behavior change:
An in-depth qualitative analysis of pregnant women and their families in Uttar Pradesh, India

Neha R. Jhaveri^1^, Natalia E. Poveda^2,3^, Shivani Kachwaha^4^, Dawn L. Comeau^1^, Phuong Nguyen^5^, Melissa F. Young^2,3^

^1^Behavioral, Social and Health Education Sciences, Rollins School of Public Health, Emory University, Atlanta, GA, USA

^2^Doctoral Program in Nutrition and Health Sciences, Laney Graduate School, Emory University, Atlanta, GA

^3^Hubert Department of Global Health, Emory University, Atlanta, GA, USA

^4^Program in Human Nutrition, Department of International Health, Bloomberg School of Public Health, Johns Hopkins University, Baltimore, MD, USA

^5^Poverty, Health and Nutrition Division, International Food Policy Research Institute (IFPRI), Washington, DC, USA

*** Correspondence:**Melissa F. Young, PhD

Emory University

Hubert Department of Global Health Department

1518 Clifton Road, Atlanta, GA

404-727-1529

[melissa.young@emory.edu](mailto:melissa.young@emory.edu)

# Supplementary Figures and Tables

**OSM Figure 1: Sampling strategy for maternal nutrition study in Uttar Pradesh**^1^

**
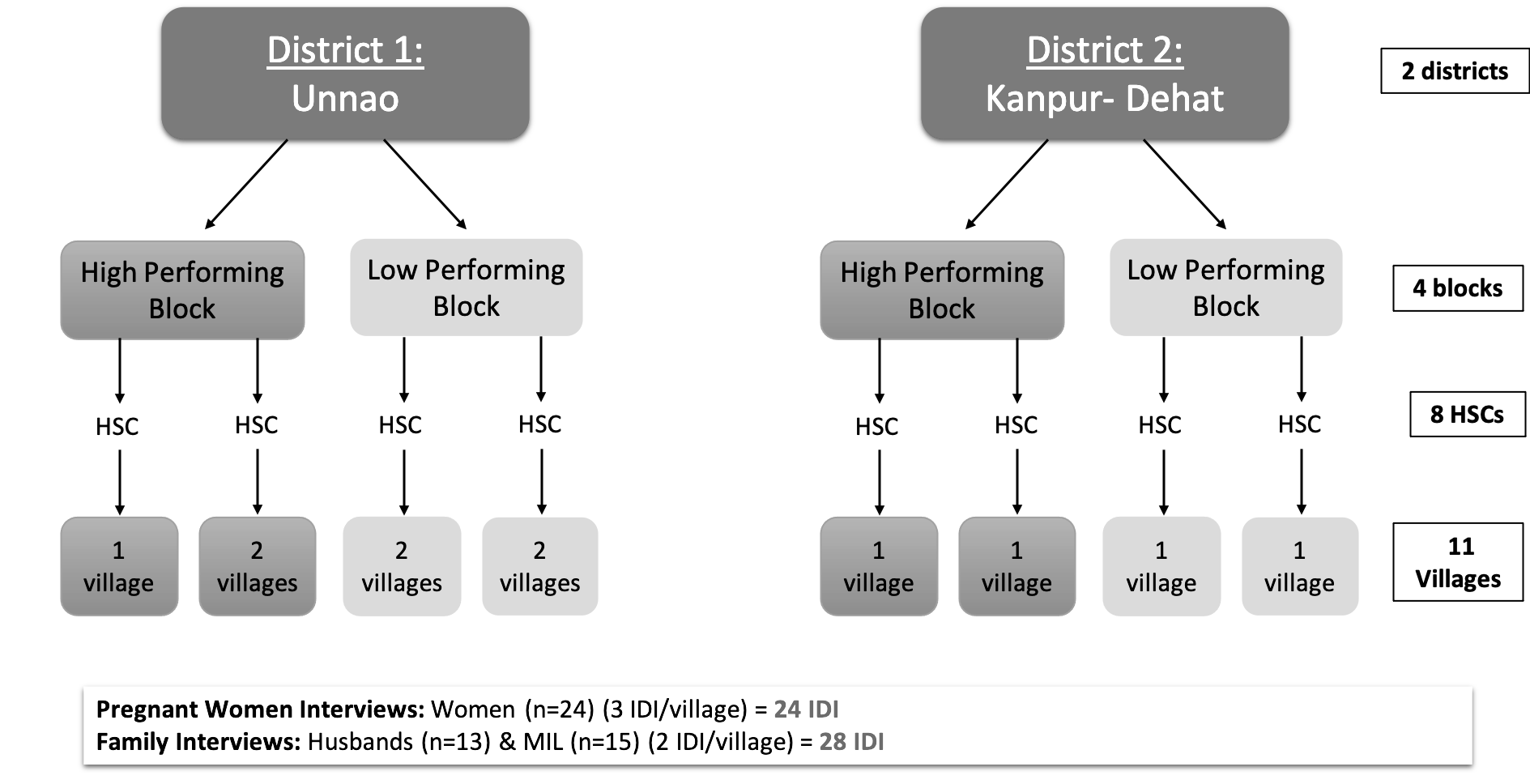
**^1^HSC= Health subcenter; IDI= In-depth interviews

**OSM Table 1: Pregnant Women In-depth Interview Guide**

| **Background Data** | |
| --- | --- |
| Age: | Did you register your current pregnancy? |
| Married (years): | How many children you have: |
| How far along in pregnancy? | Expected due date: |
| **Opening Questions** | |
| - How long have you been married? - How far along in pregnancy are you? - Who is in the household with you? - What is your routine like every day? - Are you planning on going to your mother’s place for delivery? | |
| **Pregnancy** | |
| 1. How did you feel about your health at the beginning of your pregnancy?    1. *Do you feel any changes in your health from the beginning of your pregnancy until now? If yes, could you please describe the change?*    2. *What do you think contributed to these changes?* 2. If you registered your pregnancy, when did that take place? Who did you register your pregnancy with? | |
| **Antenatal Care (ANC) and FLW Interactions:** | |
| 1. In your village, can you describe the ANC services available to you? 2. After you became pregnant, have you approached anyone to look at both your health and the health of your baby?    1. *If so, who have you been going to for seeking care?*    2. *Where is this site located?*    3. *When was your first visit?*    4. *What advice were you provided?*    5. *What were you prescribed?* 3. Can you describe your experience accessing ANC services/ advice?    1. *Where have you visited previously for ANC services during this pregnancy?*    2. *How much did you know about this location and/ or caregiver before you got to the ANC site?*    3. *What made you choose to seek care from this location and/or caregiver?*    4. *What problems did you face when accessing ANC services, if any?*    5. *What are your thoughts on the environment and the facilities provided?* 4. Can you describe the quality of your ANC visit(s) at the health center during your current pregnancy?    1. *When was your first visit?*    2. *Who, if anyone, came with you to your first appointment?*    3. *How often have you received ANC services?*    4. *What services did you receive at your last visit?*    5. *During your visit, what were you prescribed?* 5. Can you describe the quality of your ANC visit at the VHSND?    1. *What topics were discussed?*    2. *What caregivers did you interact with during your visit?*    3. *What were you given during your visit?* 6. Have you received a home visit? Can you describe the care you received at that time? 7. Have you met with an ANM? How many times? What care did you get from them?    1. *Did they take your weight? What did you learn about why gaining weight is important for you and your baby?*    2. *Did they mention the foods you should be eating while pregnant? What foods did they recommend?*    3. *Did you receive IFA?*        1. *What did they say about why it is important that you take these tablets and the directions to take the tablets?*       2. *If the ANM did not provide the IFA, who did you receive the tablets from?*    4. *Did you receive Calcium tablets?*        1. *What did they say is important that you take these tablets, number of tablets to take, and directions for how you should take it?*       2. *If the ANM did not provide the Calcium tablets, who did you receive the tablets from?* 8. During your time with the ANM, please share your thoughts on the quality of your visit.    1. *In what ways did they respond to your individual concerns?*    2. *Can you describe how they answered your questions on pregnancy and infant care?*    3. *Can you share how you felt about the clarity of their explanations on what practices you should be doing and how you should be doing it?*    4. *How easy or difficult it was to follow their advice, and why?* 9. Did you meet with an ASHA? How many times? What care did you get from them?    1. *Did they check your weight on the MCP card?*    2. *Did they check to see whether you were consuming IFA and Calcium, or if you did not have any tablets, contact an ANM so that you receive tablets afterward?*    3. *Did they discuss the meals you had recently and how you can improve your food intake? What did they share with you about this?*    4. *Did you learn about breastfeeding after giving birth? Can you describe the information you received on feeding your baby?* 10. During your time with the ASHA, please share your thoughts on the quality of your visit.     1. *In what ways did they respond to your individual concerns?*     2. *Can you describe how they answered your questions on pregnancy and infant care?*     3. *Can you share how you felt about the clarity of their explanations on what practices you should be doing and how you should be doing it?*     4. *How easy or difficult it was to follow their advice, and why?* 11. Did you meet with an AWW? How many times? What care did you receive from them?     1. *Did they provide food supplements? What did they say of the importance of consuming those?*     2. *Did they check to see whether you were consuming IFA and Calcium, or if you did not have any tablets, contact an ANM so that you receive tablets afterward?*     3. *Did they discuss the meals you had recently and how you can improve your food intake?*     4. *Did you learn about the importance of breastfeeding after giving birth? Can you describe the information you received on the importance and benefits of breastfeeding your baby?*         1. *How have your views towards breastfeeding changed throughout your pregnancy?* 12. During your time with the AWWs, please share your thoughts on the quality of your visit.     1. *In what ways did they respond to your individual concerns?*     2. *Can you describe how they answered your questions on pregnancy and infant care?*     3. *Can you share how you felt about the clarity of their explanations on what practices you should be doing and how you should be doing it?*     4. *How easy or difficult it was to follow their advice, and why?* 13. What information have you been given about feeding your child?     1. *Can you share what information was provided on how children should be fed in the first hour after birth? First month? First 6 months? From six months to two years of age?*     2. *How has the information provided changed how you plan on feeding your child after birth?* 14. How did the advice given to you differ from recommendations from family and friends? 15. Have your dietary practices changed from the beginning of pregnancy until now? If so, what did you notice? | |
| **Reflection** | |
| 1. Can you share if/how the advice provided by the caregiver during your ANC visits has changed from your first visit until your most recent visit? 2. What role did family members, friends, and community members have when practicing the advised nutritional behaviors during your pregnancy?    1. *Can you tell me how your husband is involved with your care during your pregnancy?*    2. *Can you tell me how your mother-in-law is involved with your care during pregnancy?*    3. *How has their involvement changed throughout your pregnancy?* 3. Of all the practices/ advice provided, what advice was easiest to practice routinely? What was the hardest to practice routinely, and why?      1. How has your food intake changed from before you were pregnant until now? | |
| **Closing** | |
| 1. Is there anything that you are worried about with you or your child’s health? What else would you like to know more about your health and your child’s health that was not discussed during your checkups or visits?      1. Would you like to tell me anything else about your pregnancy, or any concerns you have overall?   We really appreciate you taking time to speak to us today! | |

**OSM Table 2:** Family Members (Husbands/Mothers-in-Law) **In-depth Interview Guide**

| **Background Data** | |
| --- | --- |
| Age: | Education: |
| Relation to pregnant woman: | Occupation: |
| **Opening Questions** | |
| - Husbands: How long have you been married? - How many people live in your household? | |
| **Knowledge of maternal nutrition and breastfeeding, Awareness and perception on maternal nutrition and breastfeeding, Practices to care pregnant women / Attitude toward gender roles** | |
| 1. What do you think a woman should eat during pregnancy? 2. *How does this vary from a non-pregnant women?* 3. *Why is good nutrition important for pregnant women?* 4. *What aspects do you consider important?* 5. What have you heard about how a woman should eat during pregnancy? What counseling or information about diet for pregnant have you received? 6. *What types of foods?How many meals?How much food?* 7. *Was this information valuable/helpful? Why/why not?* 8. *Was she able to adopt these practices? Why/why not?* 9. *What difficulties (Name of woman) faced to adopt the recommended practices?* 10. *What strategies or solutions (advice) did you apply to help her to adopt the recommended practices?* 11. *What information was most useful? Why?* 12. *Which information was least useful? Why? Any recommendations you disagree with?* 13. In addition to food, are there any supplement or medicines a pregnant woman should consume? 14. *Why? Why do you think these medicines are important?* 15. *What are the benefits?* 16. *Are there any side effects? What are those?* 17. *Has [Name of pregnant women] had any difficulties taking these medicines (supplements)? Can you describe them?* 18. What counseling or information about medicines (supplements) for pregnant women have you received? 19. *Intake of IFA tablets* 20. *Intake of calcium tablets weight gain* 21. *Was this information valuable/helpful? Why/why not?* 22. *Was she able to adopt these practices? Why/why not?* 23. *What difficulties (Name of woman) faced to adopt the recommended practices?* 24. *What strategies or solutions (advice) did you apply to help her to adopt the recommended practices?* 25. *Was information was most useful? Why?* 26. *Which information was least useful? Why? Any recommendations you disagree with?* 27. Once a woman is pregnant, what activities should be done in terms of health care? 28. *ANC check-ups* 29. *Weight gain, weight monitoring throughout pregnancy* 30. What counseling or information about health care for pregnant women have you received? 31. *ANC check-ups* 32. *Weight gain, weight monitoring throughout pregnancy* 33. *Was this information valuable/helpful? Why/why not?* 34. *Was she able to adopt these practices? Why/why not?* 35. *What difficulties (Name of woman) faced to adopt the recommended practices?* 36. *What strategies or solutions (advice) did you apply to help her to adopt the recommended practices?* 37. *What information was most useful? Why?* 38. *Which information was least useful? Why? Any recommendations you disagree with?* 39. Do you think a woman need special care or attention during pregnancy? Why? Why not? 40. *What type of special care or attention does a pregnant woman need?* 41. *Who is providing support and care to (Name of women) during her current pregnancy*? 42. *Whose responsibility is it that (Name of women) have a good nutrition/health during pregnancy?* 43. *How have you helped or supported (name of women) during her pregnancy to have a good nutrition/health?* 44. What do you think children under 2 years old should eat/drink? 45. *Children in the first hour of life, in the first month, in the first six months, and between 6 months and 2 years* 46. *What are the benefits of breastfeeding?* 47. *Why is it important?* 48. *How should a child be fed that is sick?* 49. What counseling or information about nutrition for children under 6 months have you received? 50. *Breastfeeding, importance, benefits, timing* 51. Whose responsibility is it that (Name of woman) be able to breastfeed? 52. *How can you help (Name of woman)`s in her breastfeeding practice and experience?* 53. *Can you describe what are the barriers (difficulties) that (Name of woman) might experience in relation to breastfeeding?* 54. *What strategies/solutions or advice would you give to help her in the breastfeeding practice?* | |
| **Exposure to program services/ Community or social mobilization /Community groups** | |
| 1. Did you or your wife/daughter/daughter in law receive any information from AWWs, ASHAs or ANMs? 2. Can you tell me about what kind of services or information related to maternal nutrition did AWWs, ASHAs, or ANMs offer during the current pregnancy of your wife/your daughter or your daughter in-law? 3. *ANC check-ups* 4. *Interpersonal counselling about diet* 5. *Information about weight gain and monitoring* 6. *Counselling and provision of IFA pills* 7. *Counselling and provision of calcium pills* 8. *Information about breastfeeding* 9. *Knowledge of FLW* 10. *Where did you receive these services?(Home visits, AWC, Subcentre, Community events, VHNDs)* 11. What was the service or informative activity that you enjoyed the most? Why? 12. What is your experience with Ratri Chaupal? Prompt: Evening community meeting facilitated by an NGO (name of IPE Block coordinator) where husbands of pregnant women and other community members gather to discuss about maternal nutrition. 13. *Have you heard of it?* 14. *How you participated in it? If yes, how many times?* 15. *What activities are carried out during a Ratri Chaupal meeting?* 16. *What were the messages that you heard from the video and discussion? What do you think about these messages?* 17. *What kind of things did you learn about maternal nutrition and breastfeeding in this event?*   *Mothers or mothers in law   1. Can you describe what type of community groups there are in your village? 2. *Knowledge and participation in community groups such as Self-help groups (SHG)* 3. *What information is discussed in these groups?* 4. *Is there any information related to maternal nutrition? If yes, can you describe what type of information is discussed? Is it useful? Why/why not?* 5. *What are the difficulties/facilitators of attending and participating in these community groups?*   *Husbands   1. Can you describe what type of community groups there are in your village? 2. *Are there any groups for men in your village?* 3. *If yes, do you participate in these groups? Why? Why not?* 4. *What are the difficulties/facilitators of attending and participating in these community groups?* | |
| **Closing** | |
| 1. What recommendations do you have for delivery of maternal nutrition services in your family? 2. Do you have any additional comments to share before we end the interview?   Thank you very much for your time and valuable insights! | |

**OSM Table 3: Summary of the COM-B components for each maternal nutrition indicator**^1^

| **Capability** | **Opportunity** | **Motivation** | **Behavior** |
| --- | --- | --- | --- |
| ▲**▽** | ▲**▽** | ▲**▽▽** | Antenatal care checkups |
| ▲▲▲ **▽** | **▽▽** | ▲**▽** | Dietary intake |
| ▲**▽** | ▲**▽** | **●** | Weight gain/  Weight monitoring |
| ▲▲**▽** | ▲▲▲ | ▲**▽** | Iron and folic acid supplement intake |
| ▲ | **▽▽▽** | ▲ | Calcium supplement intake |
| **▽▽▽** | **●** | ▲**▽** | Intentions to breastfeed |

▲= presence of facilitators for behavior change; **▽**= presence of barriers to behavior change; **●**= not present in data.

^1^Intensity of the presence of barriers and facilitators across the interviews is noted with: one triangle=low-level; two triangles=mid-level; three triangles=high level. The intensity of the presence of barriers and facilitators reflected how prevalent the theme was across the interviews as well as the perception of how important the factor was for driving behavior change. Table represents a subjective summary of the data derived by the qualitative research team consensus in an attempt to condense the large amount of qualitative data to help inform prioritizing actions. Note: Tables 1-6 provide further depth on key themes and quotes to support overall summary.

**
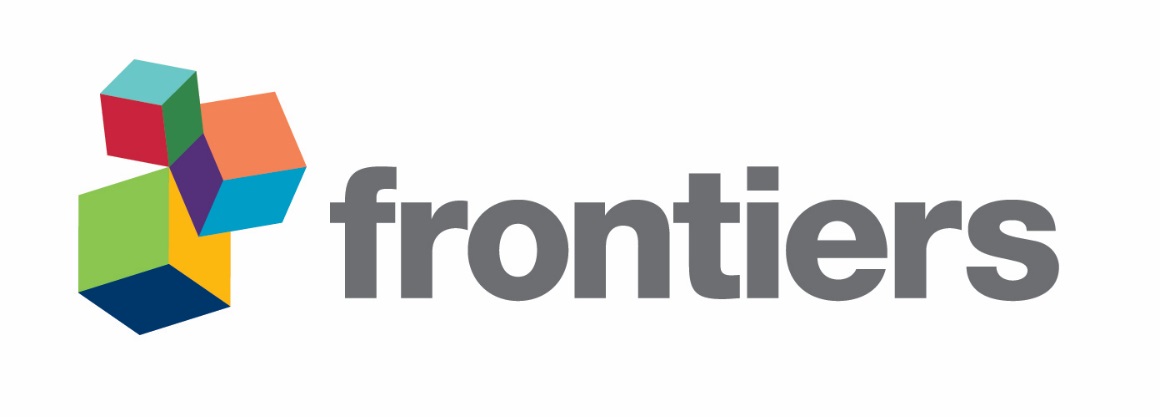
**
